# Subcutaneous Rhytidhysteron Infection: A Case Report from South India with Literature Review

**DOI:** 10.7759/cureus.2406

**Published:** 2018-04-02

**Authors:** Nagaraja Mudhigeti, Rashmi Patnayak, Usha Kalawat, Spoorthy Rekha C Yeddula

**Affiliations:** 1 Department of Microbiology, Sri Venkateswara Institute of Medical Sciences; 2 Pathology, IMS & SUM Hospital; 3 Department of Pathology, Sri Venkateswara Institute of Medical Sciences

**Keywords:** rhytidhysteron, dematiaceous fungus, subcutaneous fungal infection, molecular typing of rhytidhysteron

## Abstract

Rhytidhysteron is a saprophytic dematiaceous fungus which rarely infects humans. Though virtually all individuals are exposed, very few develop the disease. Only seven human cases are reported till date. The present case is the second case from South India.

A 40-year-old immunocompetent female agricultural worker, presented with a swelling on the dorsum of the right hand. Fine needle aspiration cytology (FNAC) of the swelling revealed short, thick, branched septate fungal hyphae. The isolate was moderately slow growing; grayish white colonies were observed on Sabouraud’s Dextrose Agar (SDA) slant. On further incubation, the colonies turned floccose, greyish black and the black pigment was observed on the reverse. Microscopy of lactophenol cotton blue tease mount showed thick, brown septate hyphae without any fruiting bodies. Molecular typing confirmed the isolates as Rhytidhysteron rufulum.

Identification of all clinical isolates of nonsporulating fungi to genus level is necessary to identify rare fungi infecting humans.

## Introduction

Rhytidhysteron is a saprophytic dematiaceous fungus usually associated with dead or decaying plant tissues [1]. Rhytidhysteron rufulum is a fungus reported which rarely infects humans [2-7]. Rhytidhysteron rufulum is capable of utilising different substrata, therefore, it occupies diverse habitats. The mould is a self-fertile, homothallic species; hence, they are able to maintain sexual reproduction even in the absence of compatible mycelia [8]. The fungus is predominantly seen in tropical and subtropical environments worldwide. This suggests that all individuals are exposed, though very few develop the disease. A thorough literature search revealed only seven human cases reported till date [2-7]. All the reports are from the Indian subcontinent. Here we present one more case of Rhytidhysteron in a healthy adult female. This is the second case from South India. Lower extremity was the site of infection in all other cases whereas in our case the swelling was on the dorsum of the right hand.

## Case presentation

**Figure 1:**
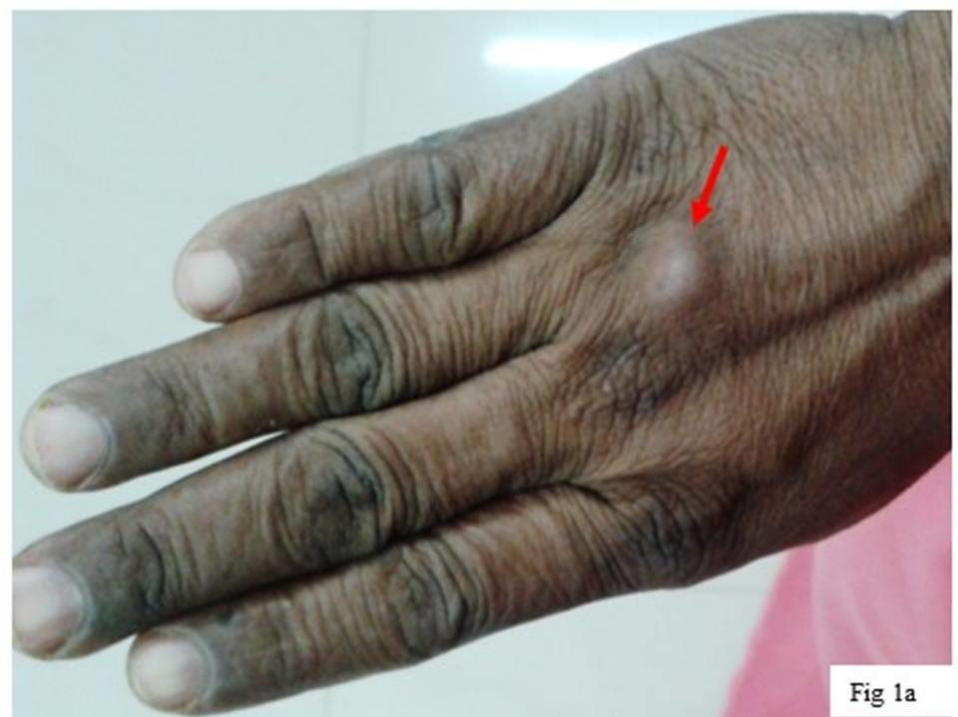
Nodule on hand. A nodule on the base of 3rd phalanx of the right hand.

A 40-year-old female agricultural worker by profession, presented with a swelling on the dorsum of the right hand. The swelling was present on the base of the 3rd phalanx of right hand since last one month. It measured 1 x 1 cm and was circular, firm, painless, and freely mobile (Figure 1).

Initially, it was small but gradually increased to present size within a span of one month. The patient was non-alcoholic, non-smoker and her blood counts and other biochemical parameters were within normal range. Her chest X-ray was normal and she was seronegative for human immunodeficiency virus. She could not recollect any history of injury related to her swelling; however, the possibility of traumatic inoculation could not be ruled out owing to her profession.

**Figure 2:**
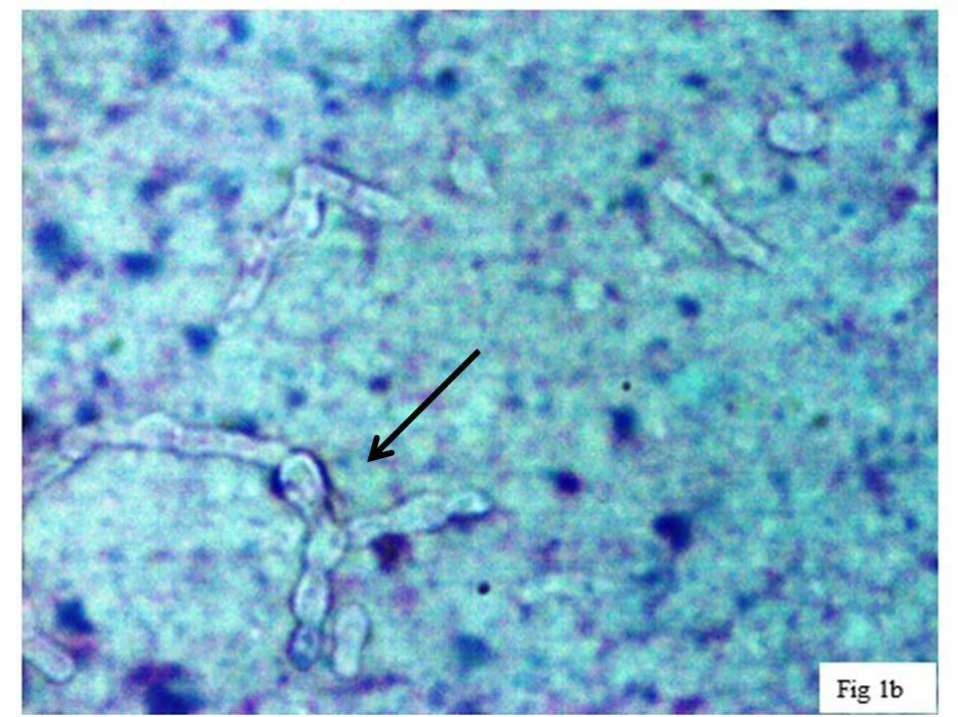
Cytology. Cytology smear showing short, thick, branched fungal hyphae with constrictions and bulbous appearance (Haematoxylin and Eosin x 400).

**Figure 3:**
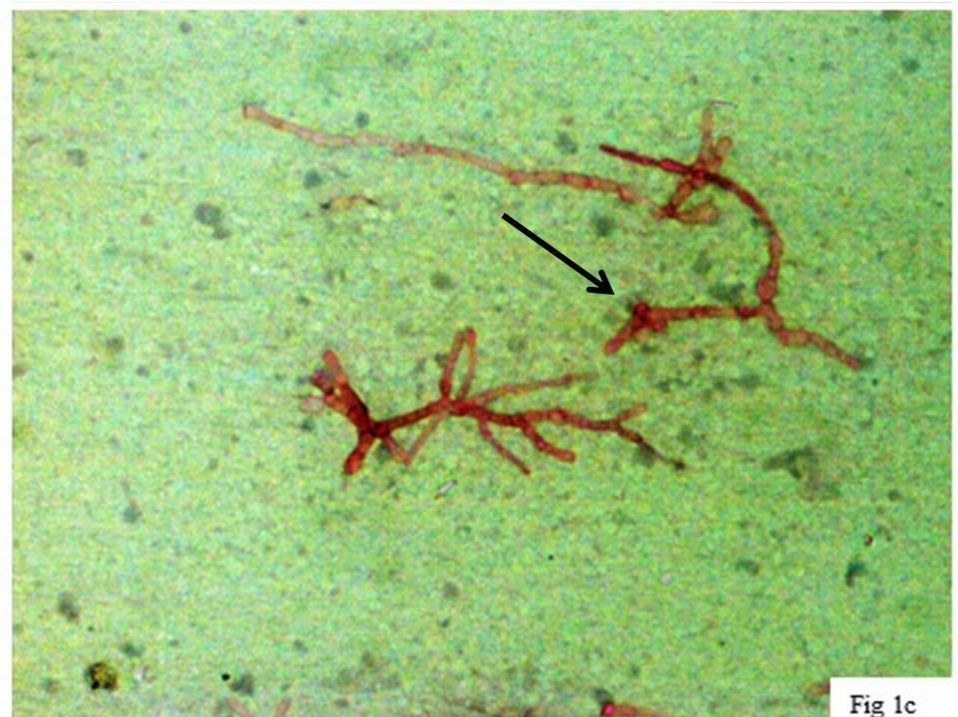
Periodic acid–Schiff (PAS) stain. Fungal hyphae highlighted in PAS stain in a background showing necrotic debris (PAS x 100).

**Figure 4:**
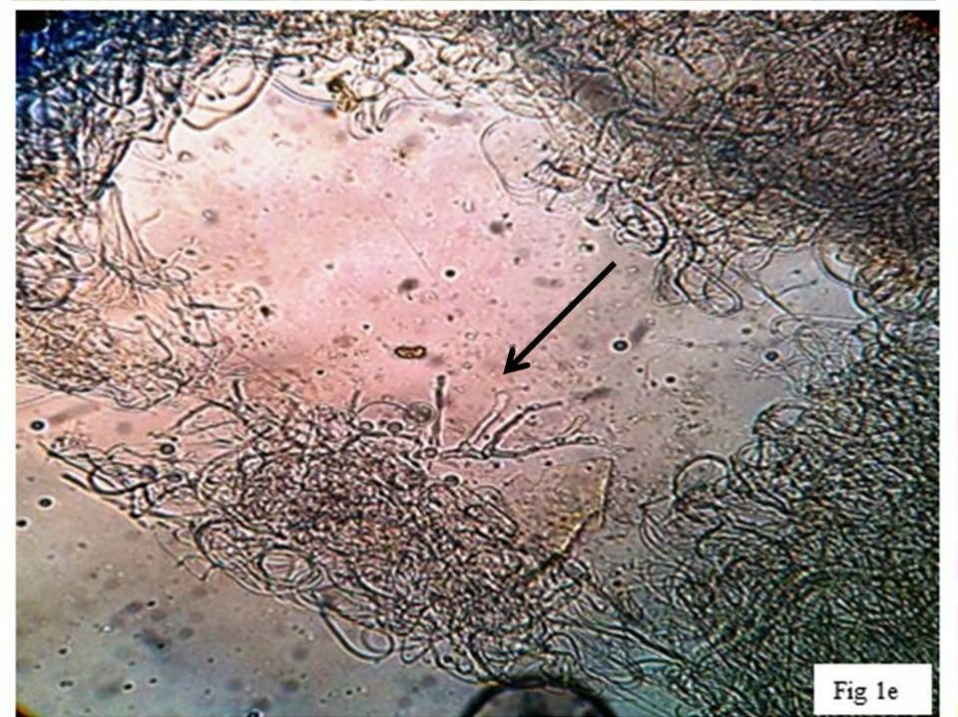
KOH preparation of the aspirate showing thin and thick-walled septate hyphae with irregular branching. KOH: Potassium hydroxide

**Figure 5:**
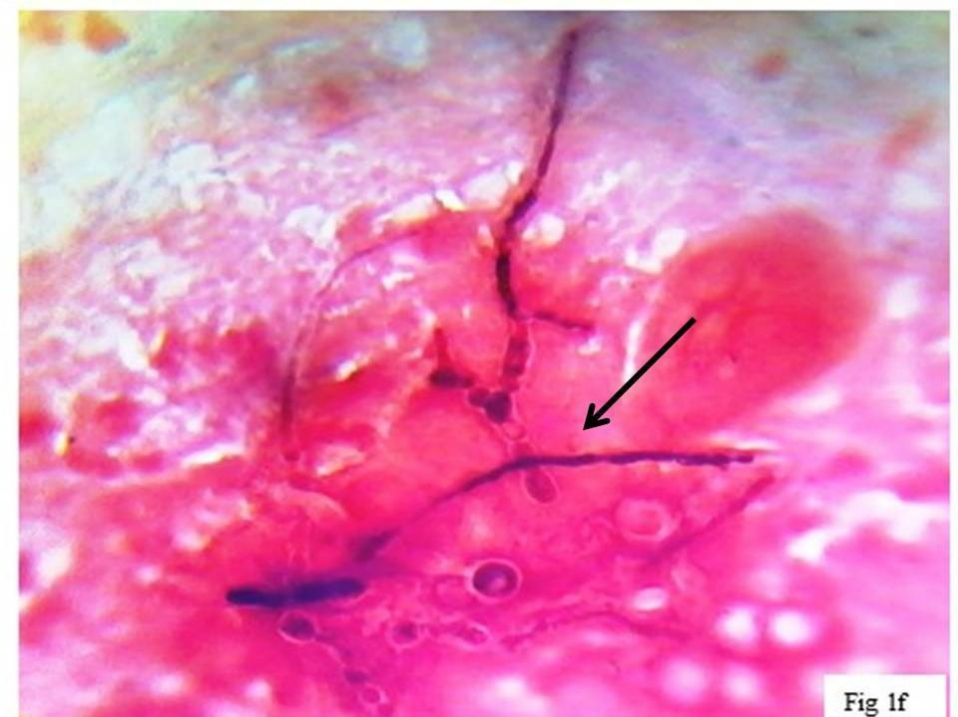
Gram-stained smear for routine bacteriology workup demonstrates short, thick, ballooning, septate hyphae with terminal chlamydospore: deeply stained (pink) cytoplasm and unstained (clear zone around hyphae) fungal cell wall is seen (x400).

Clinically it was diagnosed as ganglion and fine needle aspiration cytology (FNAC) was done. Haematoxylin and Eosin (H&E) stain of the aspirate revealed short, thick, branched septate fungal hyphae (Figure 2) highlighted on Periodic acid–Schiff (PAS) (Figure 3) and Gomori methenamine silver stain (GMS). The aspirate was also subjected to 10% potassium hydroxide (KOH) wet mount examination (Figure 4), Gram stain (Figure 5) fungal culture and molecular typing.

 

 

 

The isolate was moderately slow growing; grayish white colonies were observed after one week of incubation at 28°C on Sabouraud’s Dextrose Agar (SDA) slant. On further incubation, the colonies turned floccose, grayish black and the black pigment was observed on the reverse. Microscopy of lactophenol cotton blue tease mount showed thick, brown septate hyphae without any fruiting bodies.

The isolate failed to sporulate despite repeat attempts to induce sporulation. It was sent to Postgraduate Institute of Medical Research and Education (PGIMER), Chandigarh for molecular analysis. There genomic DNA of the isolate was amplified and sequenced using a set of primers (ITS-1, 5′-TCCGTAGGTGAACCTTGCGG-3′ and ITS-4, 5′-TCCTCCGCTTATTGATATGC-3′), which amplify the internal transcribed spacer (ITS) region of the ribosomal subunit. GenBank basic local alignment search tool (BLAST) was used to (http://www.ncbi.nlm. nih.gov/BLAST/Blast.cgi) perform sequence homology search for species identification. BLAST search confirmed the isolates as Rhytidhysteron rufulum (GenBank accession no. KJ787018). The sequence showed 98–99% homology to R. rufulum isolates in GenBank (accession no. JX868651.1, KU236376.1, KU236375.1, AM286786.1 and AM711974.1). Case management details are not known as the patient was lost to follow up.

## Discussion

Rhytidhysteron is saprophytic dematiaceous fungi usually associated with dead or decaying plant tissues [1]. No human cases were reported until recently; in 2008, one human case was reported, later in 2014, two cases and in 2016, two more cases were reported. And in 2017, two cases reported were published, one of them was reported from South India [2, 7]. Notably, six cases were reported from North India. Among these, only one case was immunocompromised. Details of these cases are summarised in Table 1.

**Table 1:** Review of published cases of Rhytidhysteron rufulum. The nodules were partially retracted in size after two weeks of therapy; however, the patient was expired due to underlying risk factors (renal transplantation, abdominal tuberculosis and multi-organ failure). PDF: Predisposing factors; DM: Diabetes mellitus; NA: Not available; GMS: Gomori methenamine silver stain; PAS: Periodic acid–Schiff.

S. No.	Case reports	Age/ gender	PDF	Site and type of lesion/nodule	Tissue reaction	Direct microscopy	Management	
							Medical	Surgical
1	Chowdhary et al. [2]	50/M	Renal transplantation	Noduloulcerative lesions on left foot and few smaller lesions over the shin and thigh	Pseudo-epitheliomatous hyperplasia with an extensive dermal infiltrate	Thick-walled, spherical, single-celled and two-celled, muriform sclerotic bodies with a brownish tinge and thick-walled chlamydospores. PAS positive.	Itraconazole	None
2	Mahajan et al. [3]	72/M	DM	Soft, painless, multi-loculated, non-tender swelling over the dorsum of the right foot with erythema and few sinuses with crusts.	Multiple areas of neutrophilic abscess bounded by epithelioid cells and foreign body giant cells	Multiple, broad, septate, irregularly branched, dematiaceous hyphae, toruloid hyphae (chains of yeast cells), and yeast-like cells which were PAS positive	Itraconazole, terbinafine and liposomal amphotericin	Swelling was surgically excised
3	Mishra et al. [4]	65/M	None	Well circumscribed, indurated, blackish, non-tender, painless subcutaneous nodule on tendoachilles region in the left foot	Intense neutrophilic reaction	Thick brown branching septate hyphae. Hyphae were PAS positive and no spherical or sclerotic bodies were present	Terbinafine and itraconazole	None
4	Chander et al. [5]	45/M	None	Mobile, non-tender swelling on thedorsal aspect of the right foot	Intense neutrophilic reaction with lymphocytes, macrophages and few septate hyphae	Long, thick, septate, tortuous, dark brown hyphae and no sclerotic bodies. Fungal hyphae highlighted on PAS staining	Itraconazole	None
5	Chander et al. [5]	50/M	None	Small, soft, non-tender, movablenodule on the anterolateral aspect of left knee			Itraconazole	Swelling was surgically excised
6	Yadav et al. [6]	54/M	None	Well-defined, painless subcutaneous swelling on anterior aspect of right leg	Acute and chronic inflammatory cells and necrotic background	Thick, long septate hyphae	Itraconazole	None
7	Tejashree et al. [7]	59/M	DM	Painless, large, soft, slowly progressive, swelling, noduloulcerative lesion on his right leg	Mononuclear inflammatory cell infiltrates	Septate, branching pheoid hyphae	Itraconazole	Swelling was surgically excised
8	Present case	40/F	None	Small, well circumscribed, firm, painless, free mobile swelling on the base of 3rd phalanx of the right hand	Inflammatory background showing neutrophils and necrotic debris	Thick branching septate hyphae with constrictions and bulbous appearance which stained positive for PAS and GMS. Occasional thick-walled chlamydospores were present	NA	NA

Rhytidhysteron rufulum is capable of utilising different substrata and occupying diverse habitats. It is seen in tropical and subtropical environments worldwide. Rhytidhysteron infects mainly through traumatic inoculation, therefore, occupation seems to play an important role in the incidence of this disease [4]. The majority of the lesions are observed on extremities of outdoor workers, mainly male rural workers. It produces primary transcutaneous lesion or swelling following several days or months of traumatic inoculation [2-7]. The size of the lesion/nodule and severity of the disease depends on the patient’s immune status and underlying risk factors such as diabetes [2-7].

To date, only seven human cases are reported in the literature, and very little is known about its pathogenicity of the mould on human cells [2-7]. All the cases reported so far have lesion in the lower extremity in contrast to the present case which was a hand swelling.

Rhytidhysteron rufulum has a putative orthologous gene set that code for dothistromin, a mycotoxin. Dothistromin is found to lyse human red blood cells (RBCs) in vitro [9]. This may explain the pathogenic potential of Rhytidhysteron on human cells.

Presentation of patients with Rhytidhysteron infection may vary from single nodule at the site of traumatic inoculation to multiple spreading noduloulcerative lesions: sometimes the overlying skin may have few sinuses with crusts [2-6]. Direct microscopy of specimens reveals thick-walled, spherical, single-celled/two-celled or brownish muriform sclerotic bodies. Sometimes thick brown hyphae with or without budding fungal bodies can be seen [2-7].

The fungus grows on any routine culture media used for isolation of fungi. Sabouraud’s dextrose agar supplemented with chloramphenicol or Potato Dextrose Agar is most commonly used media to isolate fungus from cutaneous specimens. Colonies of Rhytidhysteron appear as velvety light grey–white which floccose after one week of incubation at both 25°C and 37°C (culture tubes can be incubated at 28°C if only one tube is inoculated). The reverse of the colonies is usually brown to black. Colonies develop dark (greyish black) pigmentation on further incubation (up to four weeks) [2-7].

Lactophenol Cotton Blue wet mount of this fungus demonstrates only septate, irregularly branched, smooth-walled, sterile (vegetative) dematiaceous hyphae without any spores. Sometimes toruloid hyphae and thick-walled terminal chlamydospores can be seen. Several workers have tried to induce sporulation using different culture media including potato dextrose agar, rice agar, cornmeal agar, malt extract agar and water agar but never succeeded like the present case [2-7]. Since it is a non-sporulating mould, it is difficult to identify by the conventional methods.

Therefore, molecular characterization is an indispensable method for identification of this black mould. Molecular characterization involves amplification PCR (Polymerase chain reaction) of known conserved genes with enough sequence variation followed by sequencing and BLAST searching against GenBank or other databases for sequence homology [10]. Many authors including us have successfully identified their isolates using ITS1 and ITS4 primer set 98–100% homology with Genbank Rhytidhysteron rufulum sequences [4-7].

Patients with mild to moderate infection or uninoculated lesion respond well with oral itraconazole with or without terbinafine therapy. In contrast, patients with severe infection or widespread lesions required surgical intervention followed by prolonged combination therapy. Usually, large and multiloculated lesions require an intralesional amphotericin in addition to combination therapy [2-7]. Additional data is required to understand therapeutic options and outcome of rare Rhytidhysteron infection in humans.

## Conclusions

As Rhytidhysteron fails to sporulate in artificial culture media, PCR amplification ITS1-ITS4 genes followed by sequence analysis is the method of choice for diagnosis available at present. Identification of all clinical isolates of nonsporulating fungi to genus level is desirable to identify new/emerging fungi.
